# A Novel Audio-Perception-Based Algorithm for Physiological Monitoring

**DOI:** 10.3390/s25123582

**Published:** 2025-06-06

**Authors:** Zixuan Zhang, Wenxuan Jin, Dejiao Huang, Zhongwei Sun

**Affiliations:** 1 College of Science, Qingdao University of Technology, Qingdao 266520, China; 7711cgoswd@gmail.com (Z.Z.); huangdejiao28@163.com (D.H.); 2 College of Computer Science and Technology, Ocean University of China, Qingdao 266100, China; 22020036024@ouc.edu.cn; 3 College of Information and Control Engineering, Qingdao University of Technology, Qingdao 266520, China

**Keywords:** audio perception, NMF, respiratory monitoring, BiLSTM

## Abstract

Exercise metrics are critical for assessing health, but real-time heart rate and respiration measurements remain challenging. We propose a physiological monitoring system that uses an in-ear microphone to extract heart rate and respiration from faint ear canal signals. An improved non-negative matrix factorization (NMF) algorithm combines with a short-time Fourier transform (STFT) to separate physiological components, while an inverse Fourier transform (IFT) reconstructs the signal. The earplug effect enhances the low-frequency components, thereby improving the signal quality and noise immunity. Heart rate is derived from short-term energy and zero-crossing rate, while a BiLSTM-based model can refine the breathing phases and calculate indicators such as respiratory rate. Experiments have shown that the average accuracy can reach 91% under various conditions, exceeding 90% in different environments and under different weights, thus ensuring the system’s robustness.

## 1. Introduction

As public health awareness grows, more and more people are engaging in physical exercise. Monitoring physiological indicators is crucial for assessing individual health status and developing personalized exercise plans. Currently, smart wearable devices (such as smart clothing and smart insoles) can monitor exercise-related data, including gait and posture, thereby helping to enhance athletic performance and prevent injuries. Although a range of smart wearable devices have been developed to monitor physical activity metrics such as gait and posture, most commercial solutions do not support real-time monitoring of respiratory signals [1]. While some advanced prototypes have demonstrated potential in detecting respiratory-related metrics, these technologies remain immature or have not been widely adopted by the general consumer market. As a result, most current devices cannot reliably monitor respiration, including both the physical process of breathing (air moving in and out of the lungs) and respiratory rate (the number of breaths per minute). While breathing describes the mechanical act of ventilation, respiratory rate quantifies its frequency, and both provide distinct yet complementary insights into exercise intensity and physiological load.

Respiration, alongside heart rate, plays a vital role in evaluating physiological states during physical activity [2]. While heart rate reflects cardiovascular response and exercise intensity, respiratory patterns offer unique insights into ventilation efficiency, metabolic demand, and fatigue accumulation. For example, an abnormal breathing rhythm may indicate overexertion, ventilatory inefficiency, or insufficient oxygen uptake [3]. Therefore, combining respiratory monitoring with heart rate tracking enables a more comprehensive assessment of an individual’s physical condition and supports the design of adaptive training strategies. However, accurately capturing respiratory signals during exercise remains technically challenging due to various real-world interferences.

We propose a novel physiological monitoring approach based on in-ear audio perception. By capturing subtle physiological signals from within the ear canal, this method enables real-time and non-invasive monitoring of both respiration and heart rate. The ear canal provides a structurally enclosed and stable environment, which enhances the detection of low-frequency physiological vibrations and lays a robust foundation for our audio-based monitoring system [4].

Compared with traditional physiological monitoring methods such as photoplethysmography (PPG) or chest bands, audio-based sensing offers several notable advantages. First, it is non-invasive and does not require direct contact with the skin, avoiding signal instability caused by sweat or movement. Second, the ear canal provides a relatively enclosed and consistent structural environment, which improves the detectability of low-frequency physiological signals. Moreover, many consumer-grade earphones and headsets are already equipped with built-in microphones, making the method highly accessible and cost-effective [5]. Taken together, these advantages make audio-based measurement especially suitable for real-time monitoring during exercise.

At present, most physiological indicator monitoring solutions still rely heavily on a quiet environment, which places high demands on device performance and usage scenarios. Therefore, when respiration and heart rate monitoring are extended to sports contexts, a series of technical challenges arise. First, because the structure of the ear canal opening varies from person to person, the wearing tightness differs between users, and even the same user may exhibit inconsistent wearing conditions over time. This fluctuation in tightness directly affects the quality of signal acquisition, leading to potential errors in the system. Second, the amplitude of physiological signals related to respiration and heart rate is small and can be easily masked by other noises. During exercise, noise sources include both motion artifacts generated by human movement and ambient environmental interference, which complicates the extraction of clear and reliable physiological signals [6]. Finally, due to the lack of sufficient reference data, it remains technically challenging to segment and interpret the breathing process accurately.

Given the above problems, we propose an audio-based physiological monitoring method that uses an active noise-canceling headset to collect sound from the ear canal to monitor breathing and heart rate in real-time. This method not only fills the gap in existing respiratory monitoring but also proposes a brand-new heart rate detection method. Specifically, this study combines audio signal processing, an improved NMF algorithm, and a bidirectional long short-term memory (BiLSTM)-based respiratory detection module to extract effective physiological signals from the mixed audio generated during exercise. This method is non-invasive, portable, and real-time, providing a new solution for physiological monitoring in sports scenarios. The main contributions and innovations are as follows:

- An audio segment extraction method based on autocorrelation technology is proposed to select discrete audio segments with obvious periodic characteristics from long audio. The overall physiological state is measured by analyzing discrete audio segments that are continuous in time, which effectively overcomes the impact of individual differences and changes in wearing status on signal stability.
- Based on an improved non-negative matrix factorization (NMF) algorithm, the audio separation method can effectively separate physiological signals from noise, significantly reducing the impact of signal aliasing and noise interference.
- Based on a two-step respiratory phase detection model, it can accurately identify and classify respiratory phases, providing new ideas and solutions for developing respiratory monitoring.

The paper is structured as follows: Section 2 reviews existing physiological monitoring methods, analyzing their strengths and limitations; Section 3 introduces the system architecture, including data preprocessing, NMF-based audio separation, and BiLSTM-based phase detection. Section 4 details the improved NMF algorithm, covering signal decomposition, cost function optimization, and iterative updates. Section 5 presents the BiLSTM-based breathing model with convolutional neural network (CNN)-enhanced detection. Section 6 evaluates the NMF-based audio separation, validates the BiLSTM breathing model, and analyzes system robustness across environments, exercise intensities, body weights, and ground conditions, and Section 7 concludes with findings, limitations, and future directions.

## 2. Related Work

### 2.1. Monitoring Based on Wearable Sensor Systems

With the miniaturization of electronic devices, wearable sensor systems are widely used for health monitoring, chronic disease management, and diagnosis. Flexible sensors developed by Yasser Khan et al. [7] can monitor body temperature, heart rate, respiratory rate, blood pressure, and blood oxygen. Smart clothing and microsensors can monitor respiration and heart rate in real time [8,9,10], but usually require additional hardware, such as the Hexoskin smart clothing [11].

Wearable devices are becoming smaller and easier to use. Michelle Janusz et al. [12] used fiber optic electrodes combined with bioimpedance to detect respiratory rate. Kristin McClure et al. combined accelerometers and gyroscopes and used chest and abdominal sensors to analyze breathing patterns using a convolutional neural network. Wen Qi et al. [13] proposed a structure called WRAM, which integrates a chest strap sensor and a triaxial accelerometer to enable continuous monitoring of 15 activities.In terms of motion monitoring, the Apple Watch [14,15], Xiaomi Mi Band, and Huawei Watch can track heart rate, blood oxygen, and exercise data. For example, the Apple Watch uses photoplethysmography (PPG) technology to measure heart rate using a green LED and a photodiode [16].

However, most existing commercial devices still lack reliable and real-time respiration monitoring features, especially in dynamic and noisy environments. Therefore, non-invasive and robust solutions remain highly demanded. We propose a respiratory monitoring method based on the microphone of a smart headset, which provides a new direction for the field.

### 2.2. Monitoring Based on Audio Perception

Exercise physiological monitoring plays a crucial role in training and health management, with extensive research on audio sensing using smart headphones or wearable devices [17,18,19,20,21,22]. These methods, categorized into active and passive sensing, monitor heart rate and respiration while supporting applications like user authentication.

**Active Audio Perception:**

Active perception methods use built-in sensors and audio processing technology, where smart headphones emit specific signals through speakers, and microphones receive echo signals for analysis. A breathing monitoring system proposed by Xiangyu Xu et al. [23] collects driving environment data using acoustic devices from smartphones, employing background noise suppression and integrated empirical mode decomposition (EEMD) to extract respiratory patterns. These patterns are further processed using Generative Adversarial Networks (GAN) to generate fine-grained respiratory waveforms. Xingzhe Song et al. [24] designed the SpiroSonic system, which emits ultrasound signals through a smartphone speaker and receives chest wall reflected signals to measure the user’s lung function index.

**Passive Audio Perception:** Passive sensing processes ambient sound signals without modulation. Common placements include the ear canal, below the nose, or near the user. Kayla Jade Butkow et al. [25] used in-ear microphones to enhance low-frequency bone-conducted sounds, accurately extracting heart rate. Jagmohan Chauhan et al. [26] introduced BreathPrint, analyzing breathing patterns for user authentication with 94% accuracy. While various denoising strategies, such as empirical mode decomposition and adaptive filtering, have been proposed in prior studies, their robustness often degrades under dynamic and noisy real-world conditions.

Despite significant progress in audio perception methods, challenges remain in achieving continuous and non-invasive respiration monitoring, specifically through audio-based approaches [27]. The method proposed in this paper, using the built-in microphone of smart headphones for respiration monitoring, provides a new solution for this field.

## 3. Overview

**Figure 1:**
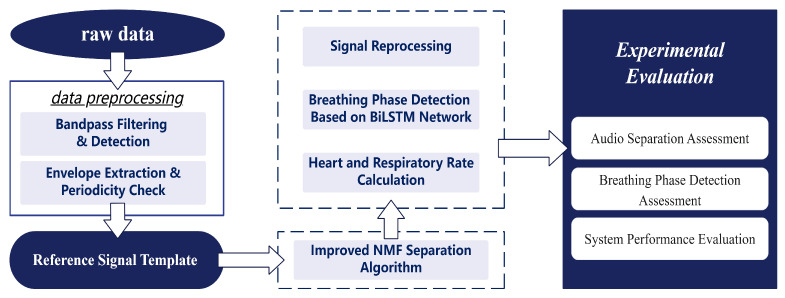
System architecture of the audio separation-based monitoring system.

We propose a physiological signal monitoring system based on the built-in microphone of active noise-canceling headphones, which acquires physiological signals such as respiration and heartbeat as well as motion noise by collecting sound from the ear canal. The system consists of data preprocessing, an audio separation algorithm, respiratory phase detection, respiratory frequency and heart rate calculation, and experimental evaluation, as shown in Figure 1.

The signals collected by the headset include breathing, heartbeat, and movement noise. Data preprocessing first uses bandpass filtering to remove noise and uses envelope extraction and periodicity analysis to select high-quality audio segments. In the audio separation stage, an improved NMF algorithm is combined with a standard source template matching method to separate breathing, heartbeat, and noise, and the signal is restored by inverse Fourier transform. In the signal reprocessing stage, a BiLSTM neural network is used for respiratory phase detection, which divides the respiratory signal into inhalation, exhalation, and pause, and then calculates the respiratory rate and heart rate to evaluate the intensity of exercise.

## 4. Improved NMF-Based Audio Separation Algorithm

### 4.1. Audio Data Preprocessing

**Figure 2:**
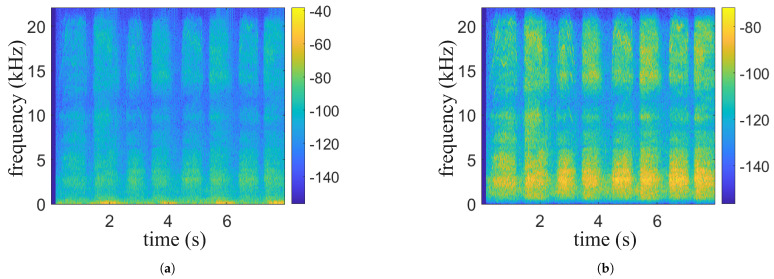
Comparison of spectrograms before and after filtering. (**a**) Raw spectrogram. (**b**) Spectrogram after band-pass filtering.

The collected audio data contains environmental noise, motion noise, and the user’s own noise, of which the environmental noise is usually above 8000 Hz. To remove these interferences, the system uses a fourth-order Butterworth bandpass filter, as shown in Figure 2. Experimental verification has shown that the physiological signal is strongest in the 500 Hz to 8000 Hz band, so this frequency band is set as the filter range. The autocorrelation function is used to detect the periodicity of the audio, while the two-threshold endpoint detection algorithm uses short-term energy and short-term zero-crossing rate to split the signal.

We propose a new endpoint detection algorithm to detect audio segments with a specific period. The specific process is shown in Algorithm 1. The short-term zero-crossing rate threshold and the high and low energy thresholds (the high threshold determines the beginning of the signal and the low threshold determines the end) are set based on the signal template. After bandpass filtering, the data is divided into 10 ms frames. If the energy exceeds the high threshold and the short-term zero crossing rate exceeds the empirical threshold for five consecutive frames, the first frame is taken as the start point, and the end point is set after 15 s to form an alternative audio clip.
**Algorithm 1** Audio Preprocessing Procedure**Require:** 
Raw audio signal**Ensure:** 
Processed audio segments or discard decision 1:**for** each audio segment $p_{j}\in P$ **do** 2:   Apply bandpass filtering to raw audio 3:   Apply dual thresholds for segment start and end points 4:   Extract envelope and downsample using Hilbert transform 5:   Calculate periodicity using autocorrelation function 6:   **if** Periodicity $\in [d_{1},d_{2}]$ **then** 7:       Output: Candidate Audio Segment 8:   **else** 9:       Discard10:   **end if**11:**end for**

**Figure 3:**
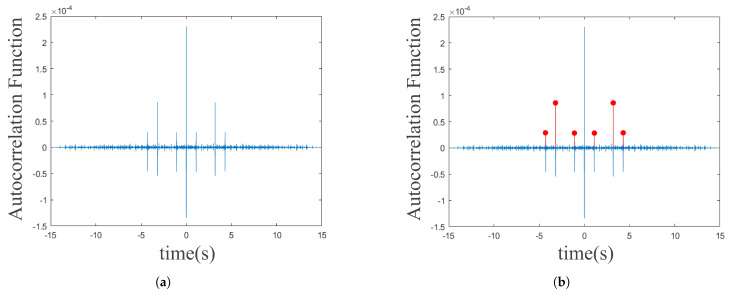
Autocorrelation analysis of audio signals. (**a**) Raw autocorrelation output. (**b**) Peaks identified by periodicity templates.

The Hilbert transform is then used to extract the signal envelope and the autocorrelation function is used to calculate the periodicity. The result is shown in Figure 3. To reduce the amount of computation, the envelope signal is downsampled to retain the periodicity information. The peak position of the autocorrelation function corresponds to the signal period, but noise can interfere with the signal period. Therefore, the average spacing of the peak positions is calculated as the period length. If the period length is within the interval [d1, d2] (determined by the template signal), the audio clip is passed to the next stage; otherwise, it is discarded.

The peak closest to zero delay is selected, and its distance to adjacent peaks is used to estimate the period *T*, calculated using Equation (1). If *T* is within [0,3] s, the candidate segment proceeds to the next step.

(1) $$\hat{T}=\frac{\mathrm{mean}\left(\mathrm{diff}(\mathrm{top}\_\mathrm{locs})\right)}{f_{s}}$$

where the diff function computes the difference between the vector elements, top_locs represents the peak positions in the autocorrelation function, and the period of the signal is estimated by averaging the differences and dividing by the sampling rate $f_{s}$.

To ensure the high quality of the input signal, only clips with a manual score of 4 or higher (on a 5-point scale) can be selected as reference signal templates. For more information on the data collection process, see the corresponding section on data collection.

### 4.2. Audio Separation Algorithm

After data preprocessing, there is a need to separate the active components from the audio signal and remove the noise. Traditional methods (empirical modal decomposition, wavelet transform) perform poorly when dealing with high-frequency overlapping signals such as breathing, heartbeat, and bone conduction sounds. Therefore, an improved NMF audio separation algorithm is proposed in this section.

The dataset used in this section is described in the Experimental Setup subsection (Data Acquisition) of the Experimental Evaluation section.

The time-frequency representation of the audio signal is denoted as V and can be obtained by the STFT, as shown in Equation (2). Perform NMF on *V* and distinguish different components, as shown in Equation (3). The goal of audio separation is to extract the breathing and heartbeat sounds from the mixed audio.

(2) v(f,t)=b(f,t)+h(f,t)+m(f,t)+o(f,t)

where v(f,t) represents the time-frequency representation of the mixed audio signal, which includes all components. b(f,t) is the breathing signal, h(f,t) is the heartbeat signal, m(f,t) is the body movement noise, and o(f,t) is the external environmental noise.

(3) $$\begin{array}{cc} V & \approx \hat{v}=WH\\ \; & =\left[W_{b}\;W_{h}\;W_{m}\;W_{o}\right]{\left[\begin{array}{cccc} H_{b} & H_{h} & H_{m} & H_{o} \end{array}\right]}^{T}\\ \; & =V_{b}+V_{h}+V_{m}+V_{o} \end{array}$$

where $W_{b},W_{h},W_{m},W_{o}$ are the basis matrices of the NMF for the different signals, $H_{b},H_{h},H_{m},H_{o}$ are the corresponding activation matrices, and $V_{b},V_{h},V_{m},V_{o}$ are the time-frequency representations of the different audio signals.

**Figure 4:**
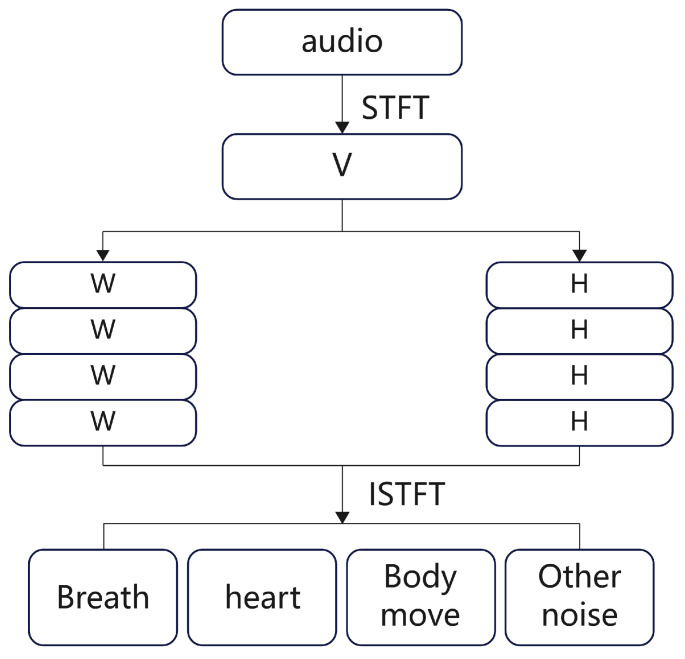
Audio separation into physiological components.

Figure 4 illustrates audio separation: the signal undergoes STFT, followed by NMF to obtain basis matrix *W* and activation matrix *H*, and is then reconstructed via inverse Fourier transform.

#### 4.2.1. Cost Function

The basis matrix *W* is categorized by signal type: $W_{1}∼W_{b}$ represents breathing, $W_{b+1}∼W_{b+h}$ represents heartbeat, $W_{b+h+1}∼W_{b+h+m}$ represents body movement, and the remaining components are classified as noise. The activation matrix *H* records the temporal activity of each signal source.

(4) $$W,H_{\min}=\arg\min_{W,H}D\left(V|WH\right)$$

(5) $$D\left(V|\hat{W}H\right)=D_{\beta }\left(V|\hat{W}H\right)+\mu {∥H∥}_{1}$$

The minimization of the error represented by WH is achieved by minimizing the cost function *D* (Equation (4)). As shown in Equation (5), the KL divergence is selected as the cost function, where the parameter β=1 represents the KL divergence. A L1 sparsity penalty (control parameter μ) is imposed on *H* to ensure that only one set of basis vectors is activated at a time, improving separation accuracy.

To separate unknown noise, an unsupervised component $W_{un}$ is introduced (Equation (6)), where $\hat{W}$ represents the signal sub-components (Equation (7)). The activation matrix *H* follows the same pattern. The hyperparameters $\lambda_{h},\lambda_{l},\lambda_{n}$ control the regularization level of the auxiliary factorization and are treated as hyperparameters.

(6) $$\begin{array}{cc} W,H_{\min} & =\arg\min_{W,H,H_{h},H_{l},H_{n}}(\lambda_{h}D\left(V|{\hat{W}}_{h}H_{h}\right)\\ \; & \;+\lambda_{l}D\left(V|{\hat{W}}_{l}H_{l}\right)+\lambda_{n}D\left(V|{\hat{W}}_{n}H_{n}\right)\\ \; & \;+D(V|{\hat{W}}_{\mathrm{un}}H_{\mathrm{un}})\\ \; & \;+\frac{1}{e_{h}}\sum_{h=1}^{e_{h}}D\left(V_{h}^{\left(i_{h}\right)}|{\hat{W}}_{h}H^{\left(i_{h}\right)}\right)\\ \; & \;+\frac{1}{T}\sum D\left(V|\hat{V}\right)) \end{array}$$

(7) $$\hat{W}=\left[\begin{array}{cccc} \frac{w_{1}}{∥w_{1}∥} & \frac{w_{2}}{∥w_{2}∥} & \ldots & \frac{w_{K}}{∥w_{K}∥} \end{array}\right]$$

where ${\hat{W}}_{h}$ represents the subcomponent corresponding to the heart rate signal, ${\hat{W}}_{l}$ represents the subcomponent corresponding to the respiratory signal, and ${\hat{W}}_{n}$ represents the subcomponent corresponding to the noise signal, with *H* also following this pattern.

#### 4.2.2. Algorithm Update Mechanism

During the reference signal template construction, this study collects pure breathing, heartbeat, and bone-conducted walking sounds for NMF iterative optimization. These signal templates are used to optimize the basis matrix $W_{b},W_{h},W_{n}$, improving signal separation performance. Based on the cost function minimization and previous equations, an iterative update method can be derived. The multiplicative update rules for *W* and *H* are shown in Equation (8):

(8) $$\begin{array}{cc} H & \leftarrow H⊗\frac{{\hat{W}}^{T}V⊗\Lambda^{\beta -2}}{{\hat{W}}^{T}\Lambda^{\beta -1}+\mu },\;\Lambda =WH\\ H & \leftarrow H⊗\frac{H_{\mathrm{num}}\left(V,W,H\right)}{H_{\mathrm{den}}\left(V,W,H\right)}\\ W & \leftarrow W⊗\frac{\left(\frac{V}{WH}H^{T}+W⊗\left(W⊗OH^{T}\right)\right)}{OH^{T}+W⊗\left(W⊗\left(\frac{V}{WH}\right)H^{T}\right)} \end{array}$$

#### 4.2.3. Implementation of the Audio Separation Algorithm

As shown in Algorithm 2, the audio separation algorithm applies the STFT to the signal, yielding the time-frequency representation *V* and phase information Phase. Specifically, STFT computes (i) *S*, the spectrogram matrix containing amplitude and phase information; (ii) *f*, the frequency vector corresponding to the rows of *S*; (iii) *t*, the time vector corresponding to the columns of *S*; and (iv) ϕ, the phase matrix. The resulting *V* is then decomposed into three components: $V_{b}$ (breathing-related), $V_{h}$ (heart rate-related), and $V_{n}$ (noise).
**Algorithm 2** Improved NMF for Audio Separation**Require:**
 Raw Audio Signal**Ensure:**
 Heart Signal and Breath Signal 1:$V_{m}$, Phase ←STFT(audio) 2:$V_{b}^{\left(ib\right)}$, $V_{h}^{\left(ih\right)}$, $V_{n}^{\left(in\right)}\leftarrow \mathrm{STFT}\left({\mathrm{audio}}_{b}^{\left(ib\right)},{\mathrm{audio}}_{h}^{\left(ih\right)},{\mathrm{audio}}_{n}^{\left(in\right)}\right)$ 3:Initialize: $H_{b}$, $H_{h}$, $H_{n}$, $H_{un}$, $H_{b}^{\left(ib\right)}$, $H_{h}^{\left(ih\right)}$, $H_{n}^{\left(in\right)}$, ${\hat{W}}_{b}$, ${\hat{W}}_{h}$, ${\hat{W}}_{n}$, ${\hat{W}}_{un}$ 4:$W=[{\hat{W}}_{b},{\hat{W}}_{h},{\hat{W}}_{n},{\hat{W}}_{un}]$ 5:$H_{m}=\left[H_{b},H_{h},H_{n},H_{un}\right]$ 6:**for** i=1 to maxiter **do** 7:   $H_{m}\leftarrow H_{m}⊗\frac{H_{\mathrm{num}}\left(V_{m},W,H_{m}\right)}{H_{\mathrm{dem}}\left(V_{m},W,H_{m}\right)}$ 8:   $H_{b}^{\left(ib\right)}\leftarrow H_{b}^{\left(ib\right)}⊗\frac{H_{\mathrm{num}}\left(V_{b}^{\left(ib\right)},W_{h},H_{b}^{\left(ib\right)}\right)}{H_{\mathrm{dem}}\left(V_{b}^{\left(ib\right)},W_{h},H_{b}^{\left(ib\right)}\right)}$ 9:   $H_{h}^{\left(ih\right)}\leftarrow H_{h}^{\left(ih\right)}⊗\frac{H_{\mathrm{num}}\left(V_{h}^{\left(ih\right)},W_{h},H_{h}^{\left(ih\right)}\right)}{H_{\mathrm{dem}}\left(V_{h}^{\left(ih\right)},W_{h},H_{h}^{\left(ih\right)}\right)}$10:   $H_{n}^{\left(in\right)}\leftarrow H_{n}^{\left(in\right)}⊗\frac{H_{\mathrm{num}}\left(V_{n}^{\left(in\right)},W_{h},H_{n}^{\left(in\right)}\right)}{H_{\mathrm{dem}}\left(V_{n}^{\left(in\right)},W_{h},H_{n}^{\left(in\right)}\right)}$11:   ${\hat{W}}_{b}\leftarrow {\hat{W}}_{b}⊗\frac{\left(\frac{V_{b}}{WH}H^{T}+W⊗\left(W⊗OH^{T}\right)\right)}{OH^{T}+W⊗\left(W⊗\left(\frac{V_{b}}{WH}\right)H^{T}\right)}$12:   $h\leftarrow \mathrm{normalization}(W_{h})$13:   ⋮14:   ${\hat{W}}_{un}\leftarrow {\hat{W}}_{un}⊗\frac{\left(\frac{V_{un}}{WH}H^{T}+W⊗\left(W⊗OH^{T}\right)\right)}{OH^{T}+W⊗\left(W⊗\left(\frac{V_{b}}{WH}H^{T}\right)\right)}$15:   $un\leftarrow \mathrm{normalization}(W_{un})$16:**end for**17:$mask_{b}=\frac{{\hat{W}}_{b}H_{b}}{WH}$18:$mask_{h}=\frac{{\hat{W}}_{h}H_{h}}{WH}$19:$V_{b}=np.multiply\left(V_{m},mask_{b}\right)$20:$V_{h}=np.multiply\left(V_{m},mask_{h}\right)$21:$audio_{breath}=\mathrm{istft}\left(V_{b},\mathrm{Phase}\right)$22:$audio_{heart}=\mathrm{istft}\left(V_{h},\mathrm{Phase}\right)$

This algorithm uses an improved nonnegative matrix factorization method to iteratively update the spectral template matrix *W* and the activation matrix *H* to effectively separate the breathing, heartbeat, and noise components in the mixed audio. The update rule in the form of a fraction comes from the multiplicative iterative algorithm, which can minimize the reconstruction error while maintaining the nonnegativity constraint, thereby obtaining a more discriminative representation of the audio components.

Since noise varies across different movement types, further refinement is required in experiments. For now, it is treated as a single category. The algorithm initializes parameters and iteratively updates the activation matrix *H* and the basis matrix *W* based on the optimization strategy and update mechanism. Additionally, an unsupervised component $V_{un}$ is introduced to handle unconsidered noise, improving signal quality. Finally, the optimized basis matrix *W* is combined with the signal template, and the breathing and heart rate audio is reconstructed via inverse STFT.

### 4.3. Physiological Indicators and Heart Rate Calculation

In strong noise, the heartbeat signal is weak and difficult to separate in a fine-grained way, so only the heart rate is calculated. An audio separation algorithm based on enhanced NMF can be used to extract respiration and heartbeat signals from mixed audio. Given the regularity of the heartbeat signal, we developed a two-threshold endpoint detection algorithm to achieve accurate signal segmentation for heart rate calculation. The data used to calculate the heart rate come from the preprocessing and separation stages, as detailed in the data collection section of the relevant part of the assessment.

**Figure 5:**
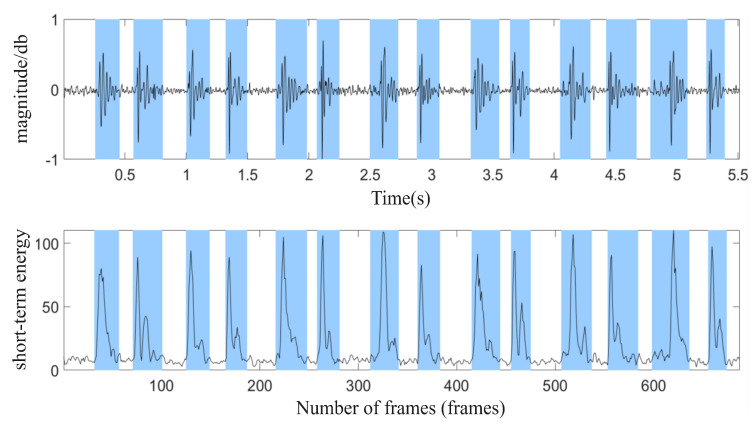
Heartbeat segmentation using short-term energy and zero-crossing rate. (**Top**): Audio waveform. (**Bottom**): Short-term energy curve of the same signal. Blue regions in both plots indicate segmented heartbeat intervals.

In this paper, the heartbeat signal is divided using short-term energy and zero-crossing rate, each heartbeat is segmented, and its start and end times are detected. The middle heartbeat is marked by a blue rectangle (as in Figure 5), the audio clip is processed, the number of heartbeats is counted, and the heart rate is calculated as the ratio of the number of heartbeats to the duration of the audio.

## 5. BiLSTM-Based Breathing Monitoring Algorithm

A complete breathing cycle can be divided into four stages: inhalation, inhalation pause, exhalation, and exhalation pause. Among these, apnoea does not need to be distinguished separately, so it is only divided into three stages: inhalation, exhalation, and pause. We propose a respiration monitoring algorithm based on a BiLSTM neural network that includes a respiration extraction module and a respiration phase detection module. It can further extract features from the audio clips with previously separated signal characteristics, accurately classify breathing phases, and thus achieve a fine-grained assessment of breathing as a physiological indicator.

### 5.1. Preprocessing and Feature Extraction

Respiratory parameters can measure the dynamic changes in breathing and can be used to distinguish respiratory audio from other sounds [28]. The core task of the feature extraction module is to extract key features to effectively distinguish between respiratory and non-respiratory audio, so not all respiratory parameters are suitable.

(9) $$mel\left(f\right)=2595\times \log_{10}\left(1+\frac{f}{700}\right)$$

MFCC (Mel-Frequency Cepstral Coefficients) is inspired by the human ear’s perception of sound. The human ear does not perceive frequencies linearly, but is more sensitive to low frequencies and relatively less sensitive to high frequencies, as shown in Equation (9). The Mel filter bank is designed to simulate this non-linear characteristic so that the MFCC is closer to the human ear’s auditory perception and thus more accurately captures the frequency distribution of the respiratory signal. Therefore, we chose MFCC and logMel as the main features to improve the representation of the respiratory signals.

After framing and applying a window to the extracted audio segments that meet the signal characteristics, a Fourier transform is performed to obtain the power spectrum, given by $P\left(f\right)=\mathrm{FFT}\left(s^{\prime }\cdot w\right)$, where $s^{\prime }$ represents the signal frame and *w* is the Hamming window. Then, P(f) is passed through a set of Mel filters to calculate the MFCC coefficients, as shown in Equation (10):

(10) $$\begin{array}{cc} M_{i} & =\log\left(\sum_{k=1}^{N}P\left(f\right)\cdot H_{m}\left(k\right)\right)\\ C_{i} & =\sum_{m=1}^{M}\alpha \left(m\right)\cdot \cos\left(\frac{\pi m(2i+1)}{2M}\right) \end{array}$$

where $C_{i}$ represents the *i*-th MFCC coefficient, $M_{m}$ denotes the *m*-th MFCC component, and α(m) is the DCT coefficient.

(11) $$\log\mathrm{Mel}\left(f\right)=10\cdot \log_{10}\left(\frac{P(f)}{1.0}\right)$$

logMel is used to compensate for the missing coefficients in the MFCC calculation, including the differential and acceleration coefficients, as shown in Equation (11). Here logMel(f) represents the logarithmic transformation of the power spectrum mapped onto the Mel scale. The denominator “1.0” is introduced to prevent zero values in logarithmic operations, thus ensuring computational stability.

### 5.2. Breathing Detection Model

We have developed a two-stage breathing detection model to achieve breathing phase detection. The model consists of two modules: the Breath Extraction Module (BEM) filters out non-breathing noise, and the Breath Phase Detection Module (BPDM) identifies different breathing phases (inhalation, exhalation, pause).

#### 5.2.1. Breath Extraction Module

The Breath Extraction Module uses a convolutional neural network to recognize segments of breath sounds and distinguish them from other sounds in the environment.

**Figure 6:**
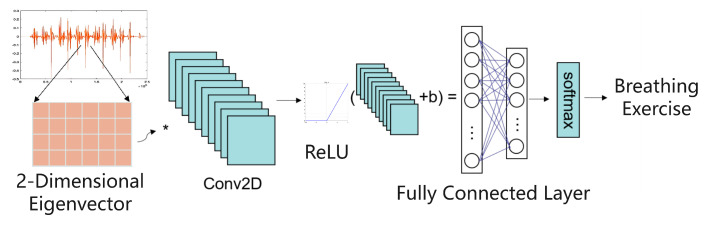
Schematic diagram of the CNN architecture for breathing classification.

We use a single-layer CNN to extract information from the acoustic features of the entire audio clip. The convolutional layer operates on the spectrogram converted from the acoustic features and extracts local patterns by multiplying the filter over the entire image. As shown in Figure 6, we apply 10 filters of size 12×12×1, use the ReLU activation function to accelerate the convergence of gradient descent, and use a fully connected layer with a softmax function to output a probability distribution.

#### 5.2.2. Breathing Phases Detection Module

In this module, we train a real-time detection model to continuously monitor the breathing phase. The system begins to detect the respiratory phase after the respiratory signal passes through the BEM module. Since the audio characteristics of the inhalation and exhalation phases are similar, it is difficult to distinguish between the two. Therefore, this paper proposes a BiLSTM neural network based on an attention mechanism to detect and evaluate the breathing phase. First, the breathing phases (inspiration, inspiration pause, exhalation, and exhalation pause) of each breathing cycle are labeled using the Viterbi decoding algorithm in the training dataset. For indistinguishable phases, the data can be discarded directly.

**Figure 7:**
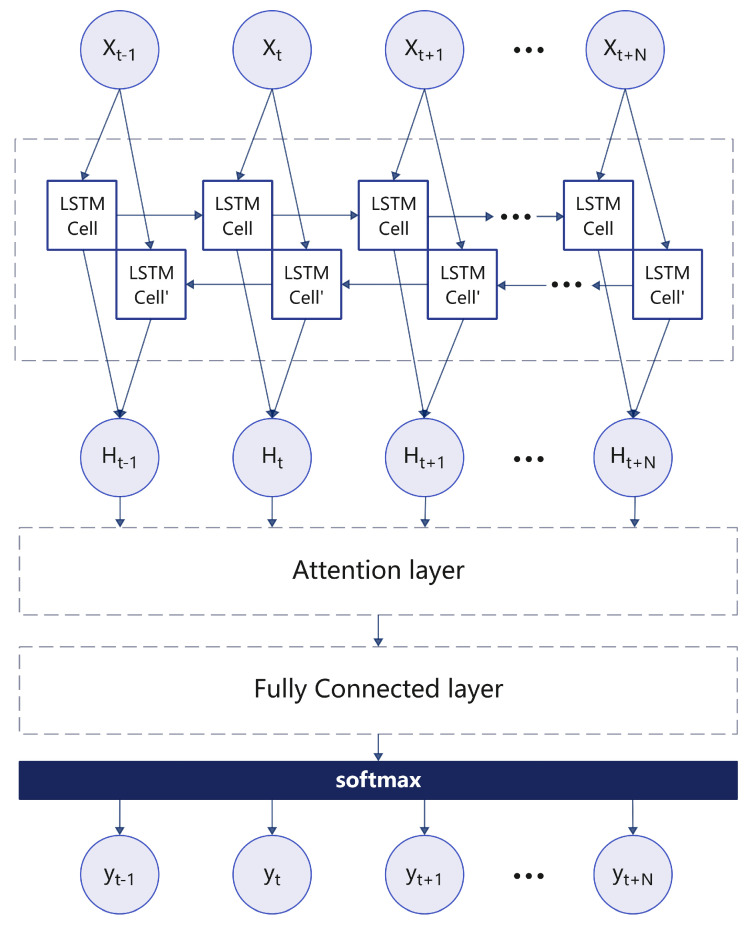
BiLSTM architecture for respiratory signal analysis.

We input each time step of the 44 ms respiratory signal. If the duration of an inhalation, exhalation, or pause is greater than or equal to 1 s, it is considered a complete breathing phase, which corresponds to a sequence of 87 samples, which is sufficient to characterize the complete breathing process. Therefore, the bidirectional LSTM (BiLSTM) architecture combines the contribution of forward units to the current hidden state and the modelling of future information by backward units, allowing the model to take full advantage of the time dependence in the entire sequence, thereby improving the modelling and analysis of respiratory signals, as shown in Figure 7.

(12) $$\begin{array}{cc} \mathrm{Attention}\;\mathrm{Score}:\;\alpha_{t,i} & =\mathrm{align}(y_{t},x_{i})\\ \mathrm{Context}\;\mathrm{Vector}:\;c_{t} & =\sum_{i=t-1}^{N}\alpha_{t,i}h_{i} \end{array}$$

The attention-based LSTM model introduces an attention mechanism to enhance the network’s memory of sequence context. As shown in Equation (12).

Where *t* represents the current time step, $\alpha_{t,i}$ denotes the attention weight of input $x_{i}$ at time step *t*, and the align function calculates the matching degree between input and output using a single-layer feedforward network. Then, the fully connected layer aggregates the output from the attention layer, and the softmax function computes the class probabilities.

The BiLSTM neural network-based real-time respiratory monitoring algorithm can continuously identify respiratory phases. By statistically analyzing the network output, respiratory ratio and respiratory frequency can be calculated.

## 6. Evaluation

### 6.1. Audio Separation Algorithms Based on Improved NMF

In this section, we evaluate an audio separation algorithm based on improved NMF, present the process of collecting the relevant data set, and analyze the quality of the separated audio and the separation effect.

#### 6.1.1. Data Collection

Breathing rate varies according to your condition. It is faster after strenuous exercise and more stable during light exercise such as walking. We therefore select the respiratory signals after running and walking to evaluate an audio separation algorithm based on enhanced NMF.

We collected breathing signals after running and after walking, using AirPods Pro to record sound via the ear canal and bone conduction. Eight volunteers participated in the experiment. Data were collected in a quiet, enclosed environment, with signal durations shown in Table 1.

- **Participants:** 8 volunteers (3 men and 5 women, aged 20–50).
- **Breathing Segments:** 100 recordings (6 min each), including:
  – 50 after walking (light breathing)
  – 50 after running (deep breathing)
- **Heartbeat Signals:** 50 recordings (60 s each) via HanHong electronic stethoscope, including 20 synchronized with breathing.
- **Bone-Conducted Sounds:** 100 recordings (6 min each) via an in-ear microphone.
- **Environmental Noise:** 50 recordings (6 min each) collected in the following:
  – Closed room
  – Gym
  – Playground

When constructing the reference signal template dataset, pure audio signals were extracted, filtered by a fourth-order Butterworth bandpass, and divided into 15 s segments. Five experts manually scored the segments based on time-frequency diagrams: 0 points indicated too much noise to detect, and 5 points indicated a clear signal with very low noise. Segments with an average score higher than 4 were used as reference signal templates.

To obtain reliable reference values for heart rate (HR) and breathing rate (BR) during exercise, we employed the Polar H10 chest strap (Polar Electro Oy, Kempele, Finland), a widely used ECG-based wearable device known for its high accuracy and robustness in dynamic conditions.

#### 6.1.2. Audio Signal Quality Assessment

We mix the breathing, heartbeat, and footstep sounds in the synchronously collected reference signal template with ambient noise such as footsteps to evaluate the audio separation effect using real values. The audio mixing uses a convolution mixing model, as shown in Equation (13):

(13) $$\begin{array}{cc} S_{\mathrm{mixture}} & =\sum_{k=0}^{3}\left(a_{\mathrm{heart}}\left(k\right)S_{\mathrm{heart}}\left(t-k\right)\right)\\ \; & \;+\left(a_{\mathrm{breath}}\left(k\right)S_{\mathrm{breath}}\left(t-k\right)\right)\\ \; & \;+\left(a_{\mathrm{noise}}\left(k\right)S_{\mathrm{noise}}\left(t-k\right)\right) \end{array}$$

where $s_{\mathrm{target}}$ represents the reference signal (heartbeat or breathing), $e_{\mathrm{inter}}$ denotes interference noise (e.g., movement noise), $e_{\mathrm{noise}}$ refers to environmental noise (background noise during recording), and $e_{\mathrm{artif}}$ corresponds to algorithmic artifact noise (introduced during signal processing or separation).

To implement convolutional mixing, three randomly generated FIR filters of length 4 are used to mix heartbeat ($S_{\mathrm{heart}}$), breathing ($S_{\mathrm{breath}}$), and noise ($S_{\mathrm{noise}}$), with corresponding filter coefficients $a_{\mathrm{heart}},a_{\mathrm{breath}},a_{\mathrm{noise}}$.

The quality of audio separation is evaluated using Signal Distortion Ratio (SDR), Signal-to-Interference Ratio (SIR), and Scale-Invariant SDR (SI-SDR):

(14) $$SDR=10\log_{10}\left(\frac{{∥S_{\mathrm{target}(t)}∥}^{2}}{{∥e_{\mathrm{inter}(t)}+e_{\mathrm{noise}(t)}+e_{\mathrm{artif}(t)}∥}^{2}}\right)$$

(15) $$SIR=10\log_{10}\left(\frac{{∥S_{\mathrm{target}(t)}∥}^{2}}{{∥e_{\mathrm{interf}(t)}∥}^{2}}\right)$$

(16) $$SI-SIR=10\log_{10}\left(\frac{\sum_{t=1}^{T}{\left(\alpha s(t)\right)}^{2}}{\sum_{t=1}^{T}{\left(s\left(t\right)-\hat{s}\left(t\right)\right)}^{2}}\right)$$

- **SDR**: Measures the ratio of signal power to distortion power between the separated and original signals (Equation (14)).
- **SIR**: Evaluates the power ratio of the signal to interference (Equation (15)), where a higher SIR indicates better signal quality.
- **SI-SDR**: Accounts for scale variations, making it more robust to scaling factors and better suited for assessing instantaneous mixtures (Equation (16)).

where s(t) is the original signal, $\hat{s}\left(t\right)$ is the separated signal, α is the scaling factor, and *T* is the signal duration.

At present, various audio separation algorithms (such as adaptive Fourier decomposition [29], empirical mode decomposition [30], and modulation filtering [31]) have achieved good results in related research. To compare the improved NMF algorithm proposed in this paper, we tested these methods under the same experimental conditions.

As shown in Table 2, the improved NMF method achieves the best SDR and SI-SIR scores, confirming its effectiveness in heart rate signal separation. While the empirical mode decomposition achieves the highest SIR, the improved NMF still surpasses other methods and approaches its performance, demonstrating its superiority.

#### 6.1.3. Evaluation of the Separation Effect

**Figure 8:**
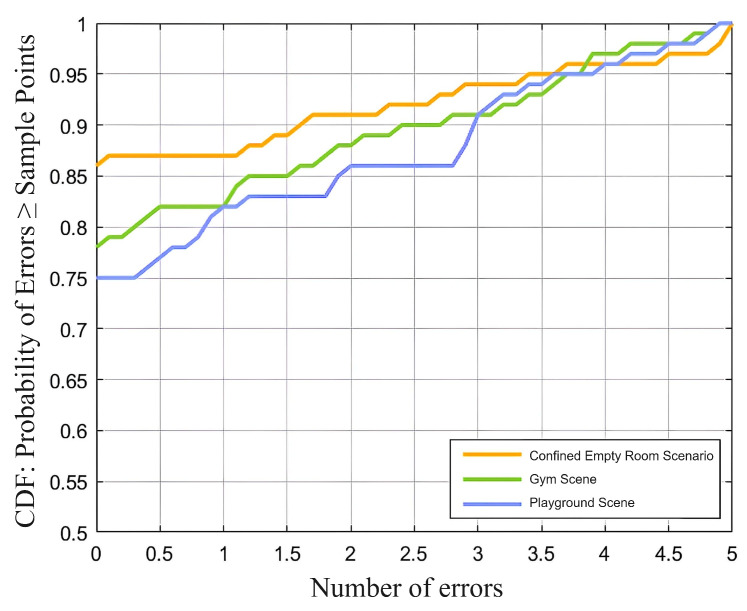
Cumulative distribution function (CDF) of respiratory rate errors in three environments. The *x*-axis represents the absolute error in respiratory rate (breaths per minute, bpm), and the *y*-axis indicates the proportion of samples with errors less than or equal to each *x*-axis value. The three environments—confined empty room, gym, and playground—represent increasing levels of background noise.

This section mixes noise with real data in three environments (closed room, gym, and playground), with a total of 100 samples. To assess whether the separated audio retains useful information and accurately reflects the breathing rate, we calculated the breathing rate and its deviation from the ground truth. Figure 8 shows the cumulative distribution function (CDF) of the error in different scenarios, indicating the system’s performance under varying noise conditions.

As can be seen in the figure, the closed room has the smallest breathing frequency error, with a proportion of 85% having an error of 0. Even in the most unfavorable situation, the proportion is still 75%, which fully confirms the effectiveness of the audio separation algorithm.

### 6.2. Breath Monitoring Algorithm Based on a BiLSTM Neural Network

#### 6.2.1. Data Collection

The improved NMF audio separation algorithm extracts breath segments based on the previous paragraph, then calculates the root mean square (RMS) at the frame level to extract the signal envelope, and uses a logical mapping to convert the original RMS to a likelihood value, as shown in Equation (17):

(17) $$p\left[S=1|RMS\right]=\frac{\exp(RMS-\tau )}{1+\exp(RMS-\tau )}$$

The decision threshold is τ=0.001, where S=1 represents inhalation/exhalation and S=0 denotes a pause. For all i,j, the transition matrix is transition[i,j]=(1−[0.5,0.6]) for j≠i, assuming inhalation/exhalation is more likely to continue, while the pause phase has equal chances of persisting or transitioning.

**Figure 9:**
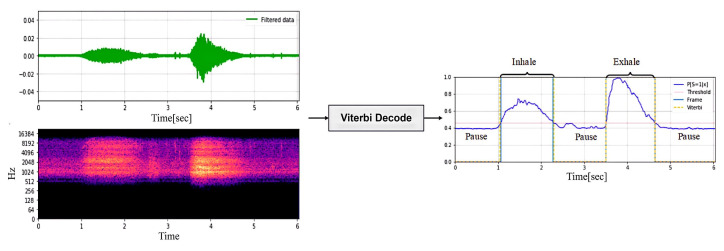
Incorrect respiratory rate in different environments.

The Viterbi algorithm [32] utilizes the given observation probability *p* and encoded transition matrix to compute the most probable state sequence (i.e., pause or inhalation/exhalation), as shown in Figure 9.

It should be noted that breath sounds vary from person to person, and the threshold τ is not a fixed value. It must be manually adjusted to optimize the cutting index. For unrecognized sounds, the electronic stethoscope recording can be used for manual labeling. A reference label is generated according to the recognized respiratory phase sequence, with the first cut point marking the start of inspiration. The labeled audio segment can be used for training.

Using this annotation method, a total of 200 groups of 15 s respiratory audio extracted by the audio separation algorithm were annotated in this paper, and an additional 50 unlabeled audio segments were added to construct the respiratory monitoring dataset.

#### 6.2.2. Comparison of the Breath Extraction Model with the Baseline Method

We compare the BEM with the baseline method using 250 data sets, of which 80% are labelled and used for training and 20% are unlabelled. ROC curves and AUC were used to evaluate the performance of the BEM method in distinguishing respiratory signals from noise, with a focus on TPR (recall rate) and FPR (false positive rate). Our method was compared with Support Vector Machines (SVMs), Random Forests (RFs), and Gaussian Mixture Models (GMMs) based on Gammatone Frequency Cepstral Coefficients (GFCCs).

**Figure 10:**
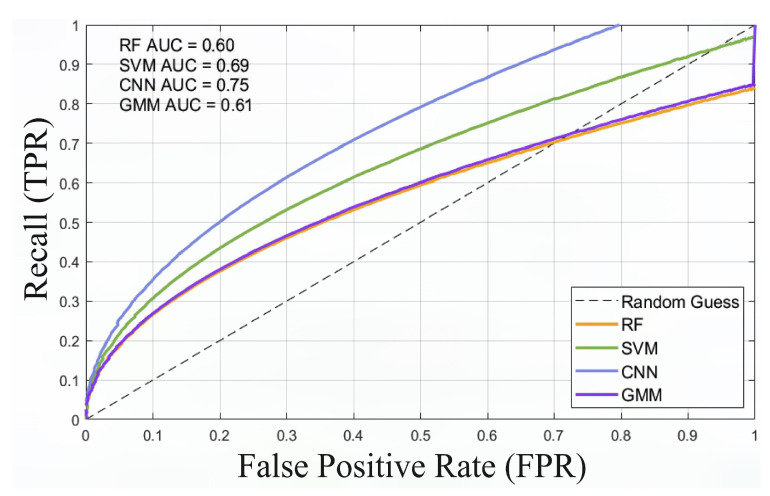
ROC curves of RF, SVM, CNN, and GMM models in respiratory sound and noise detection.

The RF, SVM, CNN, and GMM methods are used to determine whether the audio is breathing audio. The ROC curve is shown in Figure 10. For the detection of breathing sounds and no breathing sounds, the CNN method proposed in this paper performs best with an AUC of 0.75, followed by SVM (AUC = 0.69).

#### 6.2.3. Breath Phase Detection Model Evaluation

We evaluate the classification performance of the respiratory phase detection model using accuracy (ACC) and true positive rate (TPR). Additionally, we introduce an attention-enhanced BiLSTM (AttBiLSTM) model and compare its performance against the HMM model [33] and the standard BiLSTM model.

Accuracy (ACC) represents the proportion of correctly classified samples among all evaluated samples:

(18) $$\mathrm{accuracy}=\frac{TP+TN}{TP+TN+FP+FN}$$

True positive rate (TPR), also known as recall, measures the proportion of actual positive samples that are correctly identified by the classifier:

(19) $$\mathrm{recall}=\frac{TP}{TP+FN}$$

where TP (True Positive) denotes correctly classified positive samples, TN (True Negative) represents correctly classified negative samples, FP (False Positive) refers to negative samples incorrectly classified as positive, and FN (False Negative) corresponds to positive samples mistakenly identified as negative.

As can be seen from Table 3, the pause phase has the lowest accuracy and is prone to misclassification, while the exhalation phase has the highest true positive rate and more discriminable features. Comparing the HMM, BiLSTM, and AttBiLSTM models, the best TPR (0.93) and ACC (0.90) were obtained by AttBiLSTM, and HMM and BiLSTM performed similarly, suggesting that the attention mechanism helps to improve experimental performance, which will be further verified by ablation experiments in the future.

#### 6.2.4. Ablation Experiment

In this section, an ablation experiment is designed to verify the contribution of the attention mechanism added in the respiratory phase detection to the overall performance improvement of the module. The experiment compares the average prediction error of the baseline network without the attention mechanism with that of the model with the attention mechanism.

As shown in Table 4, the error of the model incorporating the attention mechanism is 2.6%, compared to 6.8% for the baseline network. These results clearly demonstrate that the attention mechanism plays a decisive role in enhancing the performance of respiratory phase detection.

### 6.3. Overall Performance Evaluation

#### 6.3.1. Data Collection

To evaluate system performance, data was collected for four exercise intensities (standing, walking, jogging, and running) in three scenarios (closed room, playground, and gym). Five floor coverings (yoga mat, carpet, wood, tile, and concrete) were tested in a closed room. Over five months, the researchers used AirPods Pro and an electronic stethoscope to record 60-s WAV audio files, collecting 5200 data sets from 10 volunteers (5 men and 5 women, aged 10–50). See Table 5 for volunteer information.

Each person collected 40 sets of data in different exercise scenarios, for a total of 4800 sets. One volunteer additionally collected 400 sets of data on floor materials. The data was divided into training and test sets in a 4:1 ratio (see Table 6) for system performance evaluation. We define jogging as 10 min/km or more, and in the experiment it was close to 10 min/km; running is defined as less than 9 min/km, and in the experiment it was close to 7 min/km.

#### 6.3.2. Experimental Accuracy Across Different Environments

**Figure 11:**
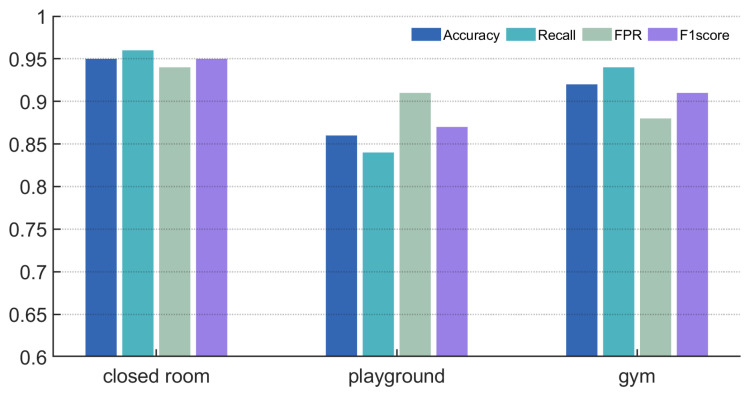
Impact of experimental environments.

As shown in Figure 11, the system has an accuracy of 95% in the closed room, indicating that the noise in an enclosed space is low and the system performs better than in an outdoor environment with the same noise level. The system uses bandpass filters to remove high-frequency noise and audio separation algorithms to remove audio aliasing noise. In playgrounds and gyms, the system’s accuracy rates were 86% and 92%, respectively, with an average accuracy rate of 91%, demonstrating its robustness in different noise environments and its effectiveness in reducing the impact of environmental noise on the monitoring of sports physiological indices, and these accuracy values were calculated as the proportion of correctly classified breathing instances among the total number of samples collected in each environment.

#### 6.3.3. Experimental Accuracy Across Different Exercise States

We divided the exercise intensity from low to high into four categories: static, walking, jogging, and running. Data was collected at all of the above experimental sites to ensure an even distribution of data. The accuracy of the experimental results is shown in Table 7. Each accuracy value in this table was computed based on the total number of breathing instances across all environments for the given exercise condition.

As shown in Table 7, the accuracy rate exceeded 89% under all four training intensities, with an average of 91.7%. When standing, due to the absence of foot bone conduction noise interference, respiratory and heart rate signals were optimally extracted, achieving an accuracy rate of 96%. The accuracy rate during jogging (93.6%) was higher than that during walking (90.4%). The results indicate that as exercise intensity increases and breathing deepens, the earplug effect enhances respiratory monitoring capability. The lowest accuracy rate was observed during fast running (85.9%), due to irregular breathing and excessive noise, which increased detection difficulty. Multiple sounds need to be suppressed to restore physiological signals.

These accuracy fluctuations under different exercise conditions reflect the challenges associated with robust signal acquisition in dynamic physiological states. Potential causes of inaccuracy include unstable in-ear sensor placement during rapid motion, increased environmental noise, and significant variability in breathing patterns.

#### 6.3.4. Mean Absolute Error in Different Environments and Exercise States

**Figure 12:**
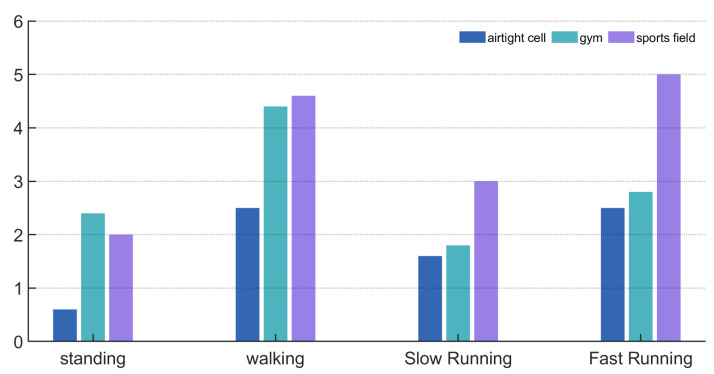
Impact of experimental environments on the mean absolute error of the respiratory rate.

We use the mean absolute error (MAE) to assess the system’s performance across different motion intensities and scenarios, as illustrated in Figure 12. MAE provides a measure of the average absolute deviation between predicted values (${\hat{z}}_{i}$) and true values ($z_{i}$), offering an intuitive evaluation of prediction accuracy, as defined in Equation (20). Here, *n* denotes the total number of breathing (or heart rate) instances used in the MAE calculation, with each instance corresponding to one paired prediction and ground truth value.

(20) $$\mathrm{MAE}=\frac{1}{n}\sum_{i=1}^{n}\left|{\hat{z}}_{i}-z_{i}\right|$$

As shown in the figure, the algorithm performs best in an enclosed space with minimal noise, with a standing respiratory frequency MAE of only 0.6. In the gym and playground, the minimum separation errors were 1 and 1.9, respectively, with an overall MAE of 2.28, showing good performance. The effect of exercise intensity on the algorithm was also analyzed, with increasing intensity in the following order: standing, walking, jogging, and running. Interestingly, jogging had a smaller error than walking, possibly because deeper breathing improved detection accuracy.

**Figure 13:**
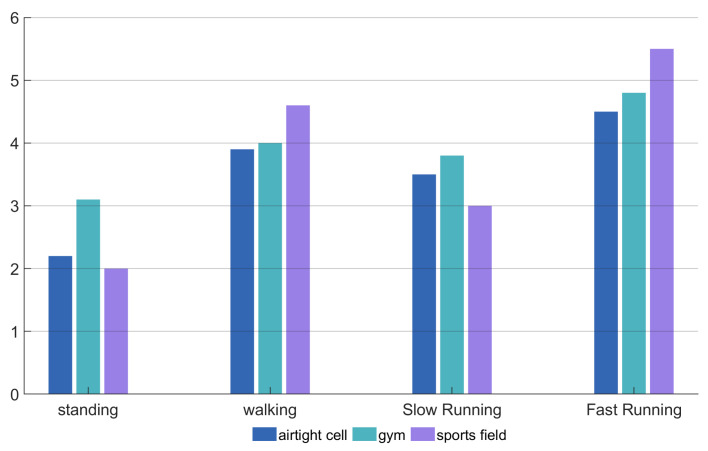
Mean absolute error of heart rate.

Figure 13 shows the heart rate MAE for standing, walking, jogging, and running in three environments. The heart rate error is greater than the breathing error, with MAEs of 2.43 (standing), 4.17 (walking), 3.43 (jogging), 4.93 (running), and a total MAE of 3.74 for mixed audio separation.

To further establish a comparative baseline, we implemented the regression-based evaluation framework proposed by Takahashi et al. [34], which utilizes linear regression with tenfold cross-validation to predict HR and BR. When applied to our dataset using Polar H10 as the reference, this method yielded mean absolute errors (MAEs) of 5.62 bpm for heart rate and 2.36 breaths/min for respiratory rate. While this demonstrates that our dataset supports reasonable estimation even under basic modeling assumptions, our proposed method still achieved notably better accuracy, with MAEs of 3.74 bpm and 2.28 breaths/min (as shown in Table 8). These results highlight the effectiveness of our signal processing approach and its robustness across diverse exercise conditions.

#### 6.3.5. Impact of Different Body Weights on System Performance

**Figure 14:**
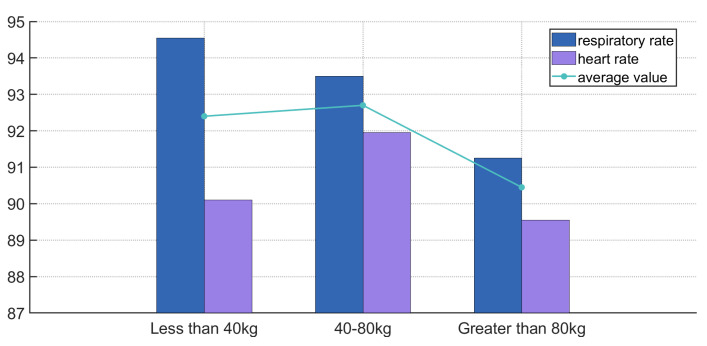
Different body weights impact the accuracy.

Body weight affects ground pressure and noise level, especially in activities with frequent ground contact like walking. Sole friction noise transmits to the ear via bone conduction and airborne pathways. Since noise is a key factor, this section analyzes its impact on results. Participants were grouped as <40 kg (2 people), 40–80 kg (6 people), and >80 kg (2 people). To prevent conscious breathing control, they were unaware of the measurement criteria. Figure 14 shows the experimental data.

As illustrated in Figure 14, both respiratory and heart rate accuracy remained stable across the <40 kg (92.4%) and 40–80 kg (92.7%) groups. Although a slight drop in accuracy was observed in the >80 kg group, the performance remained above 90%, suggesting that higher body weight may have a minor effect on signal quality. Nevertheless, the results demonstrate the system’s robustness under varying physical conditions.

#### 6.3.6. Impact of Different Floor Materials

**Figure 15:**
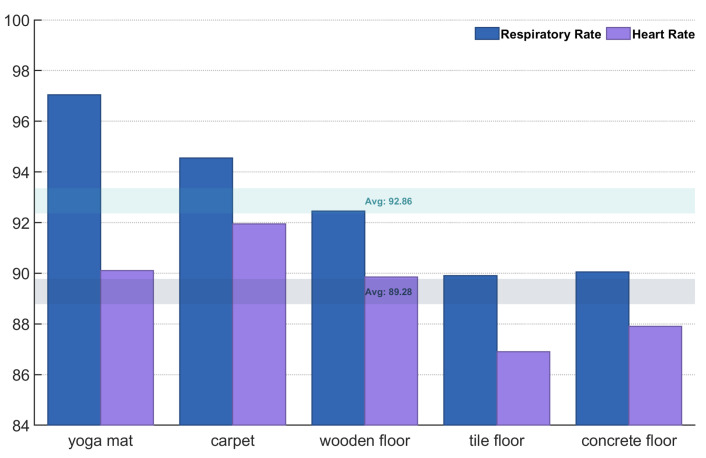
Impact of different floor materials on accuracy.

Footstep noise depends on floor material; for example, yoga mats are quieter than concrete under the same impact force. Shoe sole material also influences noise on the same floor. This experiment analyzed five floor types: yoga mats, carpets, wood floors, tiles, and concrete. 480 samples (each 15 s) were collected per floor in a closed environment, with subjects weighing 40–80 kg. Figure 15 shows the accuracy results.

As shown in the figure, yoga mats had the highest accuracy rate (97.2%), followed by carpets (94.7%) and wooden floors (92.5%), and tiles (89.8%) and concrete floors (90.1%) had the lowest. The overall average accuracy rate was 92.9%, indicating that floor material affects system accuracy, especially on tile and concrete floors.

## 7. Conclusions and Future Work

We have developed an Android app called “Hearing” that can monitor breathing and heart rate in real-time during exercise through the built-in microphone of active noise-cancelling headphones, and provide post-exercise analysis. To reduce interference from environmental and exercise noise, we propose an audio separation algorithm based on improved NMF to extract breathing and heartbeat signals from the mixed audio and reconstruct them using the Fourier inverse transform. The breathing process is divided into three stages: inhalation, exhalation, and pause. The BiLSTM algorithm is used to segment and classify the audio, thereby calculating the breathing frequency and ratio. The heartbeat signal is extracted by frame segmentation and windowing to calculate the heart rate. Compared to a single exercise index, the combination of breathing and heart rate can more accurately evaluate the exercise effect.

The system was tested in four exercise modes (standing, walking, jogging, and running) and three types of venues (indoor, playground, and gym), with an average accuracy of 91%, demonstrating good robustness and effectiveness. Future research will further optimize noise reduction algorithms, improve the quality of heartbeat signal separation, and explore the impact of multiple exercise types on physiological monitoring to enhance the system’s adaptability and stability in complex environments.

In future work, efforts will be directed toward addressing the accuracy fluctuations observed under varying exercise conditions. Specifically, improvements will focus on enhancing the mechanical stability of the in-ear sensor to reduce motion-induced signal distortion, refining signal processing techniques to better suppress environmental noise, and adapting the system to accommodate irregular breathing patterns. Additionally, future research will include expanding the experimental dataset to cover a broader range of activities and user profiles, thereby improving the robustness and generalizability of the system in diverse, real-world health monitoring scenarios.

## Figures and Tables

**Table 1:** Reference signal template used for training and validation.

Type	Duration (s)
Deep Breathing	18,000
Shallow Breathing	18,000
Heartbeat Sound	3000
Walking Bone-Conducted Sound	36,000
Enclosed Room Noise	18,000
Healthy Room Noise	18,000
Field Noise	18,000

**Table 2:** Performance comparison of audio decomposition methods.

Method	Adaptive Filter Decomposition	Experimental Mode Decomposition	Improved NMF-Based Audio Separation	Tuning Filter
SDR	11.4 dB	19.3 dB	25.5 dB	5.8 dB
SIR	15.3 dB	18.8 dB	18.5 dB	9.6 dB
SI-SIR	11.9 dB	14.2 dB	23.4 dB	7.5 dB

**Table 3:** Comparison of TPR and ACC under breathing, exhalation, and suspension conditions for three models.

Model	Breathing		Exhalation		Suspension	
	**TPR**	**ACC**	**TPR**	**ACC**	**TPR**	**ACC**
HMM	0.81	0.78	0.85	0.82	0.70	0.75
BiLSTM	0.86	0.84	0.88	0.86	0.72	0.76
AttBiLSTM	0.91	0.88	0.93	0.90	0.76	0.80

**Table 4:** Comparison of baseline network and attention mechanism models’ average prediction errors.

Baseline Network	Attention Mechanism	Average Prediction Error
✓		6.8%
✓	✓	2.6%

**Table 5:** Volunteer information statistics.

Volunteer ID	Gender	Age Group	Weight (kg)
Volunteer 1	Male	10–15	25
Volunteer 2	Male	10–15	39
Volunteer 3	Female	20–25	69
Volunteer 4	Male	20–25	71
Volunteer 5	Male	20–25	85
Volunteer 6	Female	20–25	50
Volunteer 7	Female	20–25	46
Volunteer 8	Female	20–25	52
Volunteer 9	Female	35–40	56
Volunteer 10	Male	45–50	81

**Table 6:** Data collection across different exercise scenes and locations.

Exercise Scene	Closed Room	Playground	Gym
Standing	400 sets	400 sets	400 sets
Walking	800 sets	400 sets	400 sets
Slow Running	400 sets	400 sets	400 sets
Fast Running	400 sets	400 sets	400 sets

**Table 7:** Comparison of breathing accuracy and heart rate accuracy under different exercise conditions.

Type	Breathing Accuracy	Heart Rate Accuracy
Standing	96.8	93.6
Walking	90.4	90.2
Slow Running	93.6	89.1
Fast Running	85.9	86.2
Average Value	91.7	89.2

**Table 8:** Comparison of MAE values between different methods on HR and BR.

Method	HR MAE (bpm)	BR MAE (Breaths/min)
Takahashi regression model [34]	5.62	2.36
Our proposed method	3.74	2.28

## Data Availability

The data that support the findings of this study are not publicly available due to privacy restrictions.
